# Legal Notes for Institutional Workers

**Published:** 1912-02-03

**Authors:** 


### LEGAL NOTES FOR INSTITUTIONAL WORKERS.

(The Editor will be pleased to answer Legal queries in this column.)

### A Legal Department at the Hospital.

Enough has been said to show that the poor man's lawyer does exist in England, although his powers are somewhat restricted. At any rate, to give the benefit of his counsel to the poor and needy is no breach of etiquette on the part of a member of the Bar. Many of those who attend daily at the Inns of Court have much spare time on their hands. Would it not be possible for the authorities at a large hospital to arrange for the attendance of a barrister in a room adjoining the out-patients' department for an hour or so on one or two evenings a week? There are scores of men in the Temple who would be glad to undertake the work. A slight increase of trouble to the hospital staff would be well repaid; for at the present time the house surgeon is constantly being subpœnaed as a witness in cases arising out of accidents which have brought people to the casual ward. Sound and disinterested advice would put a stop to many speculative actions. Moreover, in a *bonâ fide* case the gentleman presiding in the legal department would be in a position to recommend his "client" to a good solicitor who was willing to act. At present the "casual" is a ready prey for the lawyer's tout who hangs about the purlieus of the hospital; and the lawyers who employ these touts are no ornaments to the legal profession.

### A Hospital's Legal Rights against Noise.

Patients in hospital sometimes complain bitterly of the noises of the neighbourhood. There is much necessary noise in every crowded district; but necessary noise is seldom made the subject of complaint by those who are not hypersensitive. The rumble of traffic, the occasional shriek of locomotive or syren, the hoot of the motor horn, have less effect upon most nerves than the sound of a scratchy pen, the shout of the coal-hawker, or the dismal strains of a hurdy-gurdy. But suppose a man is hypersensitive to noise, as is the case with many invalids, will the Courts afford him protection? Could the managers of a hospital, for instance, bring suit to prevent noise being made on neighbouring premises? Some light is thrown on this question by a recent case. An artist living near Earl's Court sought to restrain the lessees from allowing noises to be made near his house. The noises complained of were the thud of an engine, the raucous voices of attendants, and the shrieks of passengers and the blare of an organ on a merry-go-round. It was alleged that the plaintiff, his wife, and child all suffered in consequence of the noise. As for the child, the judge was satisfied that it was kept awake at night and that its health had suffered. But as to the plaintiff himself, the judge said: "I am bound to hold that he has not established his case. I am not going to consider whether the artistic temperament is more sensitive than other temperaments. But it was shown that he was in a run-down condition; that the noise got on his nerves and caused a kind of mental paralysis." In the event, an injunction was granted, not because of alleged injury to the plaintiff, but because his child had suffered. It would seem to follow from this decision that patients in hospital are not entitled to any special protection from noise merely because they are unwell; and this fact makes it the more necessary for institutional managers to persuade the local authority to pass a bye-law giving special protection. A suitable form of bye-law, which prevents music and singing in any street or public place within 100 yards of a hospital, was published in a Home Office circular on January 1, 1903.

### The Goodwill of a Practice.

Certain legal questions connected with the sale and purchase of a practice are of interest to the young doctor who is embarking on a professional career and to the general practitioner who is about to retire. Is that indescribable something known as "goodwill" a marketable commodity, and, if so, who has the right to sell it? As a rule, of course, goodwill is bought and sold by means of an "introduction" for six months or so; but occasionally the purchaser of a practice has to be content to take the house of a deceased practitioner, and to avail himself of such introduction as the widow can give him. The question who has the right to sell such a goodwill has recently been discussed in the King's Bench Division. In the case in question, a practice was bought from the widow of a Dr. Corbin at nine months' purchase, it being agreed that the purchaser should board and lodge in the house and have the ordinary introduction. The question was whether the right to sell this goodwill belonged to the widow, who owned the house, or to the estate of the deceased. Mr. Justice Scrutton said: "One cannot sell a doctor's patients like slaves, or the clients of a barrister; yet there is no doubt the doctors are ready to pay some sum calculated on the profits of a doctor's business. It seems to me that they pay in respect, firstly of an unreasonable propensity in human beings to keep on going to the old house, which gives a doctor's house a certain value, and, secondly, of the fact that if people are told that B is the successor of A they will go to B for that reason. I think the plaintiff sold, first, the right to carry on business in the house which is her own, and, secondly, the right to say 'I am Dr. Corbin's successor.'" He therefore held that the widow had the right to sell the practice. It would seem that he attached importance to the ownership of the house. In his Lordship's view, the goodwill of what may have been described as a *post-mortem* practice runs with the house. How the case would have been decided if the house had not belonged to the widow must be left over for further consideration. As to the "unreasonable propensity" to which Mr. Justice Scrutton alludes, no doctor who is buying a practice can afford to ignore it.

